# Supplementary material for: Environmental Toxin Biliatresone-Induced Biliary Atresia-like Abnormal Cilia and Bile Duct Cell Development of Human Liver Organoids
Source: Toxins (Basel). 2024 Mar 11;16(3):144. doi: 10.3390/toxins16030144 (PMC10974618; doi:10.3390/toxins16030144)
Supplement: Supplementary file 1 [file toxins-16-00144-s001.zip › toxins-2870914-supplementary.pdf]

Supplementary Materials: Environmental Toxin Biliatresone-Induced Biliary Atresia-like Abnormal Cilia and Bile Duct Cell Development of Human Liver Organoids

Yue Hai-Bing [1], Menon Sudheer Sivasankaran [1], Babu Rosana Ottakandathil [1,†], Wu Zhong-Luan [1], So Man-Ting [1], Chung (Patrick) Ho-Yu [1,2], Wong (Kenneth) Kak-Yuen [1,2], Tam (Paul) Kwong-Hang [1,2,3] and Lui (Vincent) Chi-Hang [1,2,*]

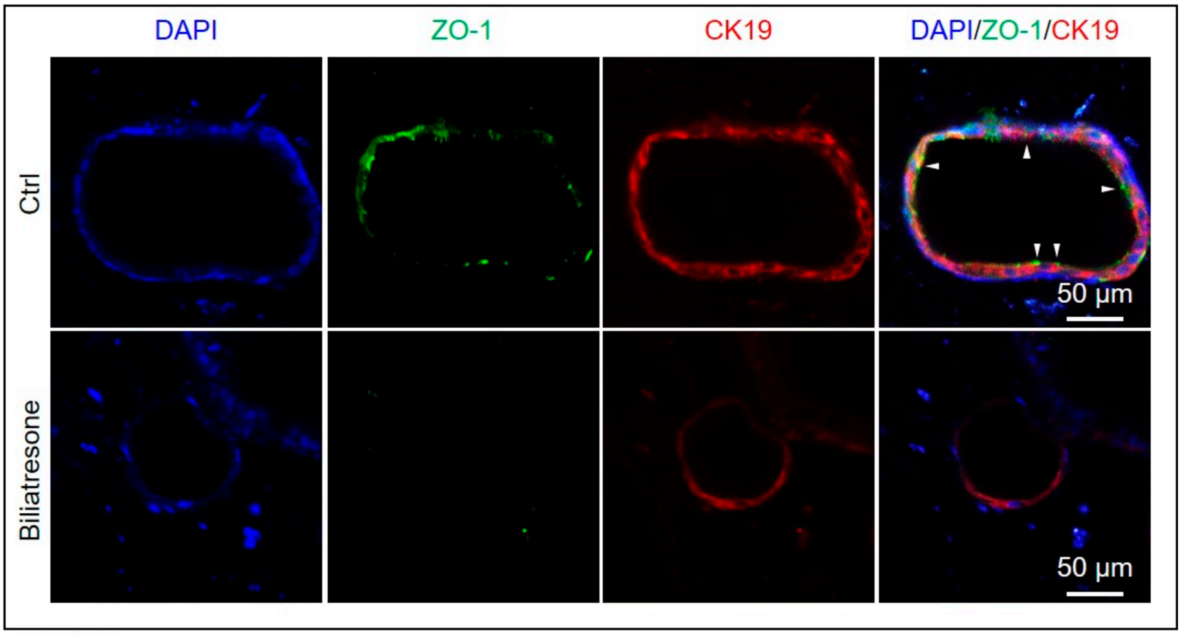

Figure S1

Figure S1. Immunostaining of control and day-2 biliatresone-treated human liver organoids for ZO-1 (green) and CK19 (red). Nuclei were stained with DAPI (blue).

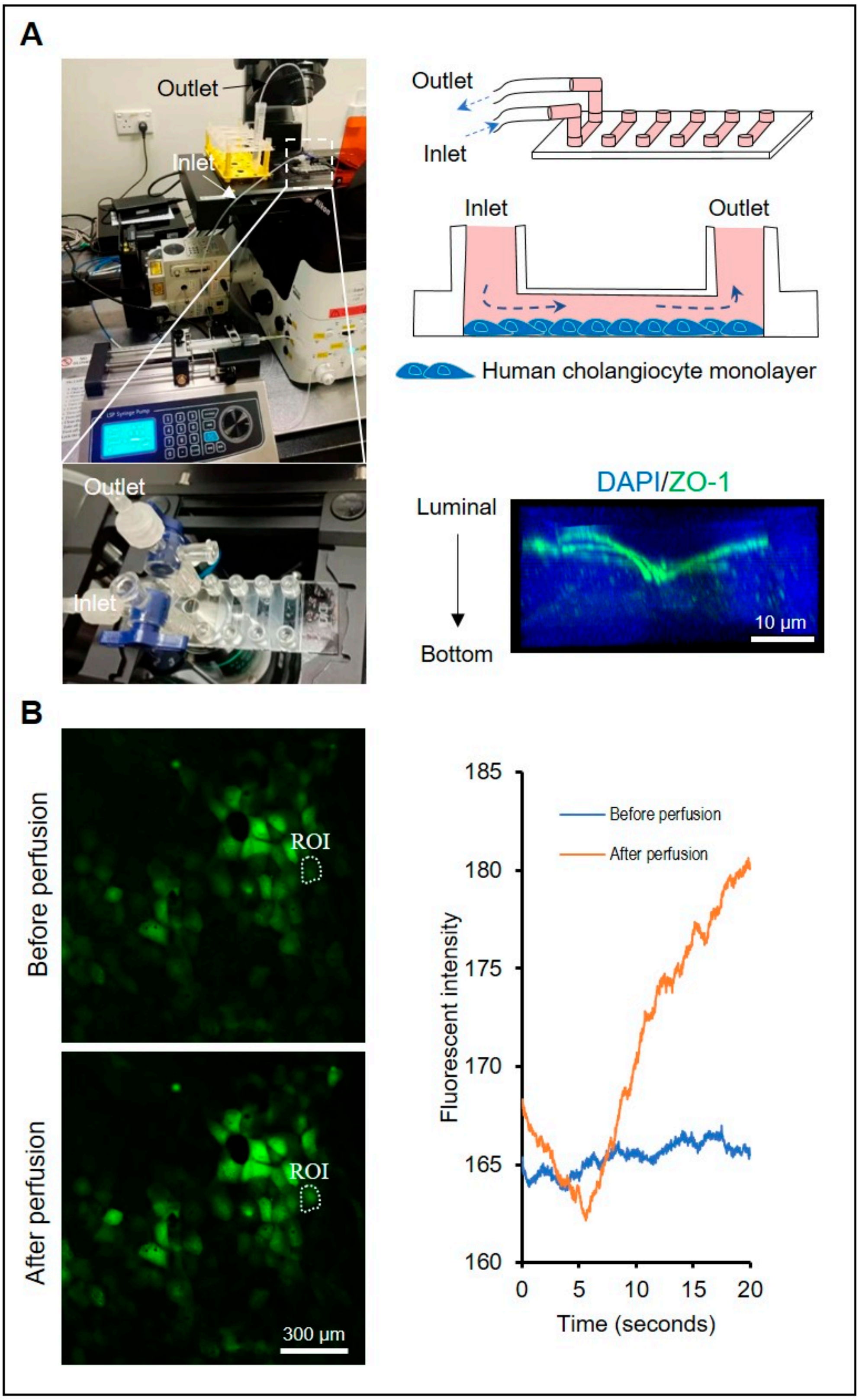

Figure S2

Figure S2. Setup of calcium signal evaluation of human cholangiocytes after perfusion stimulation. (A) Left panel, perfusion setup for the microfluidic chip mounted on the microscope. Boxed region was magnified and shown below. Right panel, diagram of the microfluidic chips with human cholangiocyte monolayer, and representative image of the cholangiocyte monolayer cultured in the channel of microfluidic chip immunostained for ZO-1 (green). Nuclei were stained with DAPI

(blue). (B) Left panel, representative images of cholangiocytes stained with Calbryte 520 before and after perfusion stimulation. Right panel, plot profile of fluorescent intensity changes of highlighted region (ROI: gion of interest) for 20 seconds after perfusion stimulation.

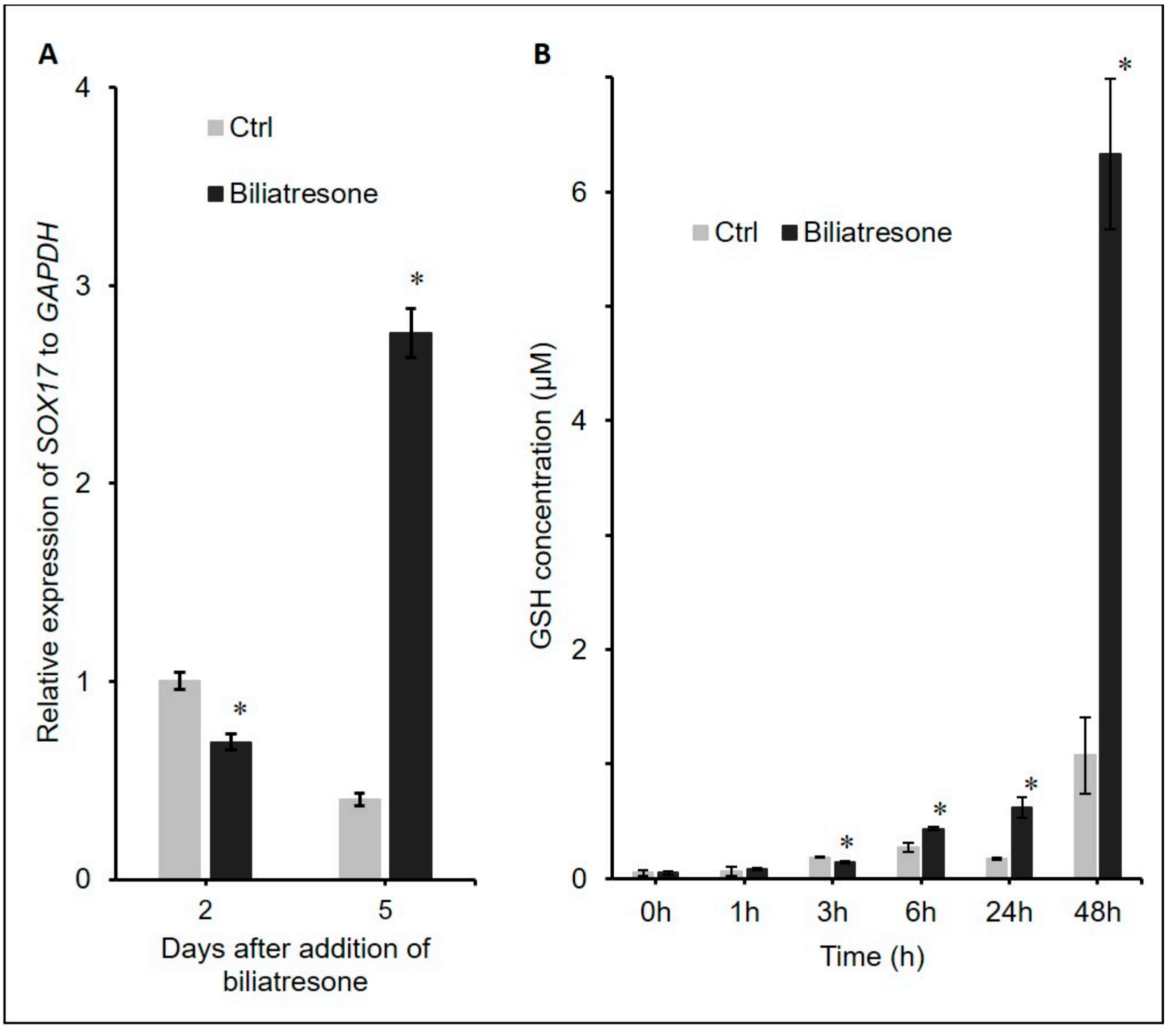

Figure S3

Figure S3. Gene expressions in liver ductal organoids with or without biliatresone treatment for 2 day and 5 days. (**A**) mRNA expression levels were characterized by real-time PCR for *SOX17* normalized to *GAPDH*. (**B**) GSH levels were measured in liver organoids from 0 to 48 hours without (Ctrl) or with biliatresone. Data was obtained from 3 wells in each group; *, p<0.05, student's t-test; error bars indicated the standard deviation.

Table S1. Patient information.

**Diagnosis**	**Age**	**Gender**	**Clinical Summary**
HB	8 mth	F	Partial hepatectomy, uneventful recovery
	15 mth	M	Partial hepatectomy, uneventful recovery
	24 mth	F	Partial hepatectomy, uneventful recovery
CC	86 d	M	Excision of extrahepatic choledochal cyst, uneventful recovery
	7 mth	F	Excision of extrahepatic choledochal cyst, uneventful recovery
	29 mth	F	Excision of extrahepatic choledochal cyst, uneventful recovery
	34 mth	M	Excision of extrahepatic choledochal cyst, uneventful recovery

Movie S1:

(https://hkuhk-
my.sharepoint.com/:v:/g/personal/vchlui_hku_hk/EZO6Z1DLtdpLnIZAV6993xUB0aywDBZjCsbSPUs6GfPuQ
g?e=aIogxf).
